# Puffing as a Novel Process to Enhance the Antioxidant and Anti-Inflammatory Properties of *Curcuma longa* L. (Turmeric)

**DOI:** 10.3390/antiox8110506

**Published:** 2019-10-23

**Authors:** Yohan Choi, Insu Ban, Hyungjae Lee, Moo-Yeol Baik, Wooki Kim

**Affiliations:** 1 Department of Food Science and Biotechnology, Graduate School of Biotechnology, Kyung Hee University, Yongin 17104, Korea; yo3247@naver.com (Y.C.); qksdlstn@naver.com (I.B.); 2 Department of Food Engineering, Dankook University, Cheonan 31116, Korea; lee252@dankook.ac.kr

**Keywords:** turmeric, curcuminoid, puffing, antioxidant, anti-inflammatory

## Abstract

*Curcuma longa* L. (turmeric) is used as a food spice; however, its strong taste restricts wider applications as a food ingredient despite its well-known health benefits. To develop an effective yet simple process for enhancing its antioxidant and anti-inflammatory activities, turmeric was gun-puffed at various pressures. Puffed turmeric exhibited an increase in its brown color and porous structures, indicating the occurrence of the Maillard reaction and vaporization during the process. Proximal analysis revealed that puffing did not alter the major constituents, although a very small decrease in crude fat extraction was observed under some circumstances. Total phenolic compounds in the extract were significantly increased after puffing, and subsequent assessment of antioxidant capacity, as determined using independent 2,2-diphenyl-1-picrylhydrazyl (DPPH), 2,2’-azino-bis (3-ethylbenzothiazoline-6-sulphonic acid) (ABTS), and ferric reducing antioxidant power (FRAP) assays, demonstrated enhanced antioxidant capacity in a puffing-pressure-dependent manner. Turmeric extract was further tested for the regulation of inflammatory responses in the murine macrophage RAW264.7 cell line. Suppression of pro-inflammatory cytokines interleukin (IL)-6 and tumor necrosis factor (TNF)-α in lipopolysaccharides (LPS)-induced macrophages was amplified using puffed-turmeric extracts compared to the control extract. Furthermore, macrophage-activation assessment revealed downregulated expression of inflammation-relevant cluster of differentiation (CD)80 and CD86 using puffed-turmeric extract in a puffing-pressure-dependent manner. However, expression of major histocompatibility complex (MHC)-II, which controls adoptive immunity, was not affected by treatment with any of the turmeric extracts. Overall, the current study demonstrated that puffing is a promising and simple method for enhancing the antioxidant and anti-inflammatory properties of turmeric.

## 1. Introduction

*Curcuma longa* L. (turmeric) is a perennial herbaceous plant belonging to the genus *Curcuma* in the Zingiberaceae family and is cultivated throughout Asia. In addition to its use as a food spice, turmeric has long been used as a traditional medicine in India [1]. Recent studies have identified its major active compounds to include 4–6% curcuminoids, 2–4% essential oils, and 2–3% fixed oils [2]. Curcuminoids, the principal pigment and bioactive compounds in turmeric, are composed of curcumin (1,7-bis-(4-hydroxy-3-methoxyphenyl-1,6-heptadiene-3,5-dione) and its derivatives demethoxycurcumin (DMC) and bisdemethoxycurcumin (BDMC), which have been widely studied for antioxidant, anticancer, antimutagenic, and antibacterial functions [3,4,5]. The major component, curcumin, is a polyphenolic compound classified as generally recognized as safe (GRAS) by the U.S. Food and Drug Administration [6,7]. Despite its health benefits, turmeric, as well as its products, are limited in food applications due to its unique flavor and pungent taste. The low stability and bioavailability of curcuminoids also restrict the use of turmeric.

Puffing, a cooking process using heat and pressure, is classified into two types: an atmospheric pressure method called oven puffing and a pressure drop method called gun puffing [8]. Both methods are based on vaporization of the moisture in the matrix of the food material; however, gun puffing induces expansion of the material through a sudden opening of a heated chamber to decrease the pressure [9]. Consequently, puffing contributes to physical changes in a solid including volume expansion, increased porosity, and decreased hardness. In addition, evaporation of water during the puffing process aids in the extension of the product’s shelf life. Chemical changes, including starch gelatinization and the Maillard reaction, noted in plant-derived foods also occur during puffing [10]. The Maillard reaction generates browning pigments and volatile substances, such as formic acid, acetaldehyde, formaldehyde, and glyoxal, which impart desirable flavors to the material [11]. In addition, puffing increases the antioxidant activity and extraction yield of bioactive compounds of doraji (*Platycodon grandiflorum*), cacao beans (*Theobroma cacao* L.), and ginseng (*Panax ginseng* C.A Meyer) [9,12,13]. Therefore, puffing is a potential treatment for various food materials, including turmeric, to improve functionality and palatability [14,15,16].

Inflammation is a complicated immune response to either exogenous irritators or internal damaged tissues. Therefore, inflammation itself is an essentially physiological component of the host defense, yet uncontrolled responses, termed chronic inflammation, are detrimental. Chronic inflammation can cause rheumatoid arthritis, psoriasis, multiple sclerosis, various cancers, and type I diabetes [17,18,19], and macrophages are key players in both the initiation and resolution of inflammation [20,21]. Therefore, many studies have attempted to regulate inflammatory responses in macrophages using various herbal extracts to prevent and/or ameliorate chronic inflammation.

Although the anti-inflammatory effects of turmeric extract have been previously reported [22], the current study sought to determine the optimal conditions for puffing to maximize the functionality of turmeric extracts. In this regard, the extraction yield and health-aiding functions of extracts of turmeric with or without puffing were examined.

## 2. Materials and Methods

### 2.1. Materials and Chemicals

Sliced and dried turmeric (cultivated in Jindo-gun, Republic of Korea, and harvested in October–December 2017) was purchased from Bibong Herb Co. (Yangju-si, Republic of Korea). Food-grade 70% ethanol was purchased from Ethanol Supplies World Co. (Jeonju-si, Republic of Korea), and methanol, sodium carbonate, sodium hydroxide, and hydrochloric acid were purchased from Daejung Chemicals & Metals Co. (Siheung-si, Republic of Korea). Folin–Ciocalteu’s phenol reagent, 2,2′-azino-bis(3-ethylbenzothiazoline-6-sulfonic acid) diammonium salt (ABTS), 2,2′-azobis(2-amidino-propane) dihydrochloride (AAPH), 1,1-diphenyl-2-picrylhydrazyl (DPPH), gallic acid, catechin, and ascorbic acid were purchased from Sigma Aldrich Co. (St. Louis, MO, USA). Aluminum chloride and sodium nitrite were purchased from Junsei Chemical Co., LTD (Tokyo, Japan).

### 2.2. Puffing Process

The puffing of turmeric was carried out in the presence of four parts by weight dried rice to prevent excessive carbonization in the high temperatures while achieving the target pressure more quickly, as previously reported [15]. Briefly, a mixture of turmeric and rice was placed in the chamber of a rotary gun puffing machine and heated. Subsequently, the internal pressure of the chamber was increased to either 686, 784, 882, or 980 kPa, at which point the chamber door was suddenly opened to induce puffing through the rapid pressure release. Non-puffed turmeric served as a control, and all samples were stored at −20 °C until the subsequent experiments

### 2.3. Proximate Analysis

The control or puffed turmeric samples were ground in a commercial blender (SFM-353NK, Shinil Industrial Co., Ltd., Cheonan-si, Korea) and sieved through a 20-mesh screen. The moisture, crude ash, crude fat, and crude protein contents were measured according to the Korean Food Standard Codex. Briefly, the moisture content was determined using oven drying at 105 °C and the crude fat content was measured using the Soxhlet extraction method. The crude protein content was calculated from the measured nitrogen content using the Kjeldahl method with 6.25 as the nitrogen–protein conversion factor. The ash content was determined from the weight of the turmeric following burning at 600 °C for 24 h.

### 2.4. Colorimetric Measurement

The color difference in ground turmeric following puffing was measured using a colorimeter (JC801, Color Techno System, Tokyo, Japan). All measurements were performed on triplicate samples. The Hunter *L*-, *a*-, and *b*-value coordinates ranged from *L* = 0 (black) to 100 (white), *a* = −80 (green) to 100 (red), and *b* = −80 (blue) to 70 (yellow). The values were calibrated with a white standard plate of *x* = 93.82, *y* = 95.67, and *z* = 113.78.

### 2.5. Extraction

A total of 200 mL of 70% ethanol was added to 5 g of ground turmeric (20:1, v/dried w), followed by stirring with a magnet at room temperature for 30 min. The extracts were vacuum filtered and the filtrate was stored at −20 °C until subsequent experiments. To determine the extraction yield, the extracts were dried at 105 °C in a drying oven (HB-502M, HanBaek Scientific Co., Bucheon-si, Korea), and the yield was calculated using:

(1) $$\mathrm{Extraction}\;\mathrm{yield}\;\left(\%\right)=\frac{\left(w_{2}-w_{1}\right)}{A}\times \frac{E}{E^{\prime }}\times 100$$

where A is the weight of the turmeric (g), E is the total volume of the extract (mL), E’ is the used volume of extract (mL), w_1_ is the initial weight of the aluminum dish (g), and w_2_ is the weight of the aluminum dish and the solid (g).

### 2.6. Measurement of Total Phenolic and Total Flavonoid Contents

The total polyphenol content (TPC) of turmeric was measured using Folin and Ciocalteu’s method [23]. Briefly, 200 μL of extract, 2.6 mL of distilled water, and 200 μL of Folin–Ciocalteu solution were reacted for 6 min. Following the reaction, 2 mL of Na_2_CO_3_ was added, and the absorbance was measured at 750 nm after 90 min. Gallic acid was employed as the standard, and the results are expressed as mg gallic acid equivalents (GAE)/g dried turmeric. The total flavonoid content (TFC) was measured according to the method from Zhishen, Mengcheng, and Jianming with a modification [24]. Briefly, 0.5 mL of diluted extract, 3.2 mL of distilled water, and 0.15 mL of 5% NaNO were mixed. After 30 min, 0.15 mL of 10% AlCl_3_ was added. After 1 min, 1 mL of Na_2_CO_3_ was added, and the absorbance at 510 nm was measured. Catechin was used as the standard and the results are expressed as mg catechin equivalents (CE)/g dried turmeric.

### 2.7. Assessment of Radical-Scavenging and Ferric-Reducing Activities

The DPPH radical-scavenging activity of the extracts was determined using the method from Brand-Williams, Cuvelier, and Berset [25], modified to use DPPH and 80% methanol to prepare a 0.1 mM DPPH solution. Briefly, the DPPH solution was first adjusted to an absorbance of 0.650 ± 0.020 at 517 nm. Next, 0.05 mL of the extract was added to 2.95 mL of the DPPH solution and reacted at room temperature for 30 min. The absorbance at 517 nm (zeroed at DPPH absorbance) was then determined. Ascorbic acid served as the standard, and the radical-scavenging activity of the extract is expressed as mg vitamin C equivalents (VCE)/g dried turmeric.

The ABTS radical-scavenging activity of the extracts was determined using a modification of the method from van den Berg, Haenen, and Bast [26]. Briefly, a mixture of 1.0 mM AAPH, 2.5 mM ABTS, and phosphate buffered saline was reacted for 30 min at 70 °C. After filtration through a 0.45-μm syringe filter, the ABTS reagent was adjusted to an absorbance of 0.650 ± 0.020 at 517 nm. Next, 980 μL of the ABTS reagent was mixed with 20 μL of extract and reacted at 37 °C for 10 min. The absorbance at 517 nm (zeroed at ABTS absorbance) was then measured.

The ferric reducing ability of plasma assay (FRAP) was performed according to the method of Benzie et al. [27]. Briefly, three solutions were prepared: 300 mM acetate buffer (pH 3.6; 3.1 g C_2_H_3_NaO_2_∙3H_2_O in 16 mL C_2_H_4_O_2_), 10 mM TPTZ (2,4,6-tripyridyl-s-triazine) solution in 40 mM HCl, and 20 mM FeCl_3_∙6H_2_O solution. Fresh FRAP solution was then prepared by mixing 25 mL acetate buffer, 2.5 mL TPTZ solution, and 2.5 mL FeCl_3_∙6H_2_O solution and was warmed to 37 °C before use. A total of 2850 mL of the FRAP solution was added to 150 mL of extract and reacted for 30 min in the dark. The absorbance of the colored product (ferrous tripyridyltriazine complex) was then measured at 593 nm.

### 2.8. RAW264.7 Cell Culture

Murine macrophage RAW264.7 cells were cultured at 1 × 10^5^ cells/well on 24-well plates in Dulbecco’s modified Eagle’s medium (DMEM, Welgene, Gyeongsan, Korea) supplemented with 10% fetal bovine serum (FBS, Welgene) and 1% antibiotic/antimycotic solution (10,000 U/mL penicillin G, 10,000 μg/mL streptomycin, and 25 μg/mL amphotericin B, Welgene) at 37 °C in a 5 % CO_2_ incubator (Model BB15, Thermo Scientific, Waltham, MA, USA). The cells were grown with or without turmeric extract for 24 h, and inflammatory responses were stimulated using 500 ng/mL lipopolysaccharide (LPS, Sigma-Aldrich Co., St. Louis, MO, USA) for 12 h.

### 2.9. Pro-Inflammatory Cytokines and mRNA Quantification

Following LPS-induced activation, the cells and culture media were separated via centrifugation at 300× *g* for 5 min. The supernatants were then analyzed to quantify the levels of pro-inflammatory cytokines (interleukin (IL)-6 and tumor necrosis factor (TNF)-α) using enzyme-linked immunosorbent assay (ELISA) according to the manufacturer’s instructions (BD Biosciences, Sparks, MD, USA). Total RNA was isolated from the cell pellets using an MG Total RNA Extraction kit (MG Med, Seoul, Korea) according to the manufacturer’s instructions. The RNA concentration and purity were assessed on a Nanodrop 2000 spectrophotometer (Thermo Scientific, Waltham, MA, USA). Transcription of the inflammatory genes mIL-6 (F: 5′-GTACTCCAGAAGACCAGAGG-3′, R: 5’-TGCTGGTGACAACCACGGCC-3’) and mTNF-α (F: 5′-CCTGTAGCCCACGTCGTAGC-3′, R: 5’-CTCAGCACCCACCCGCTCA-3’) was measured using quantitative reverse-transcriptase PCR (qRT-PCR) with a MG One-step RT-PCR MasterMix (SYBR Green) (MG Med) and a CFX ConnectTM Real-Time PCR Detection System (Bio-Rad, Hercules, CA, USA) under the following conditions: 42 °C for 10 min, 95 °C for 10 min, followed by 39 amplification cycles at 95 °C for 5 s and 55 °C for 30 s. The 2-ΔCt method was used to calculate the relative mRNA expression levels of the target genes compared to the internal control gene mRNA of glyceraldehyde 3-phosphate dehydrogenase (mGAPDH) (F: 5′-CACTCACGGCAAATTCAACGGC-3′, R: 5′-CCTTGGCAGCACCAGTGGATGCAGG-3′) using Bio-Rad CFX Manager software v3.1. Specifically, ΔCt was calculated as Ct_target gene_ – Ct_GAPDH_, where Ct represents the amplification detection cycle number of an arbitrary threshold.

### 2.10. Surface Protein Expression Analysis

Expression of surface proteins on RAW264.7 cells following extract treatment and LPS stimulation was determined using flow cytometry (Accuri™ C6, BD Biosciences, Sparks, MD, USA) with fluorescence-conjugated antibodies, as previously described [10,28]. Briefly, cells were analyzed following the gating of intact macrophages using an side-scatter (SSC) vs. forward-scatter (FSC) scatterplot. The expression of cluster of differentiation (CD)80, CD86, and major histocompatibility complex (MHC)-II on the macrophages was determined using the mean fluorescence intensity (MFI) of the fluorescence (FL)1, FL2, and FL4 channels, respectively, as assessed using BD Accuri^TM^ C6 software v1.0 (BD Biosciences) [20].

### 2.11. Statistical Analysis

All experiments were repeated at least three times. All experimental data were analyzed using one-way analysis of variance (ANOVA) and are expressed as mean ± standard deviation (SD). Duncan’s multiple range test was conducted to assess significant differences among experimental mean values for the colorimetric and antioxidant capacity analyses using SAS software, version 8.2 (SAS Institute, Inc., Cary, NC, USA). Tukey’s post-hoc multiple comparisons test was applied to cytokine and activation marker expression analyses using GraphPad Prism software, version 5 (La Jolla, CA, USA). For all analyses, *p* < 0.05 was considered statistically significant with designation using letters in an alphabetical order of the mean values.

## 3. Results

### 3.1. Alteration of Turmeric Morphology and Color after Puffing

**Figure 1:**
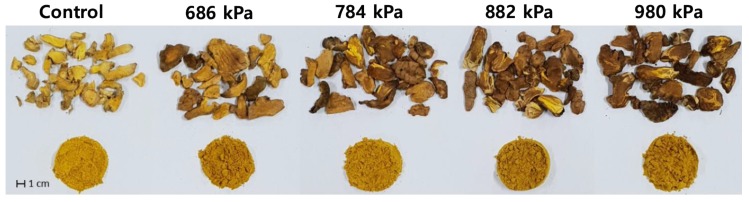
Morphological change of turmeric caused by puffing at various pressures. Darkening of the turmeric correlated with increased puffing pressure was observed in both the puffed material (upper) and ground powder (lower).

Turmeric underwent morphological changes during puffing, which were apparent in both the raw material and ground powder (Figure 1). Increased browning and expansion of the turmeric were observed as the puffing pressure increased.

**Figure 2:**
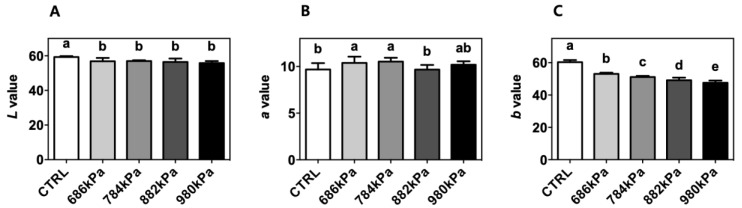
Colorimetric changes in the *L* (**A**), *a* (**B**), and *b* values (**C**) of turmeric following puffing at various pressures (*n* = 3). CTRL—control.

The colorimetric properties of the control and puffed turmeric were determined in terms of Hunter’s color space *L*, *a*, and *b* values (Figure 2). The *L* values of puffed turmeric (56.84 ± 1.91, 56.98 ± 0.43, 56.45 ± 1.96, and 55.82 ± 1.12 for 686, 784, 882, and 980 kPa, respectively) were lower compared to that of the control (59.31 ± 0.48), indicating the darkening of turmeric through browning reactions. The *b* value decreased as puffing pressure increased (60.32 ± 1.36, 53.08 ± 0.79, 51.15 ± 0.72, 49.14 ± 1.63, and 47.59 ± 1.41 for the control, 686, 784, 882, and 980 kPa, respectively). The *a* values of the turmeric powder increased after puffing compared to the control except for the sample created at 882 kPa (9.68 ± 0.69, 10.39 ± 0.67, 10.53 ± 0.41, 9.67 ± 0.49, and 10.19 ± 0.37 for the control, 686, 784, 882, and 980 kPa, respectively).

### 3.2. Proximate Analysis

The proximate compositions of the control and puffed turmeric samples are described in Table 1. Crude protein, fat, and ash contents were calculated based on the dry weight of turmeric since the moisture content was significantly affected by puffing. Specifically, the moisture content decreased in a pressure-dependent manner from 15.69 ± 0.09% for the control to 10.74 ± 1.22% at 980 kPa. The crude fat content tended to increase with increased pressure; however, only the turmeric puffed at 784 (7.46 ± 1.27%) and 882 (7.57 ± 0.69) kPa showed a significant increase versus the control (6.39 ± 0.14%). Puffing did not affect the crude protein or ash content in the turmeric.

### 3.3. Extraction Yield, Total Phenolic Content (TPC), and Total Flavonoid Content (TFC)

The extraction yields of the control and puffed turmeric samples are described in Table 2. Although no increase in total solid extraction in turmeric was observed following puffing, TPC significantly increased as puffing pressure increased (Table 2) from 8.81 ± 0.81 for the control to 13.49 ± 0.66 mg GAE/g dried turmeric for the sample puffed at 980 kPa. However, TFC exhibited no significant difference among the control and puffed turmeric samples.

### 3.4. Antioxidant Capacity

**Figure 3:**
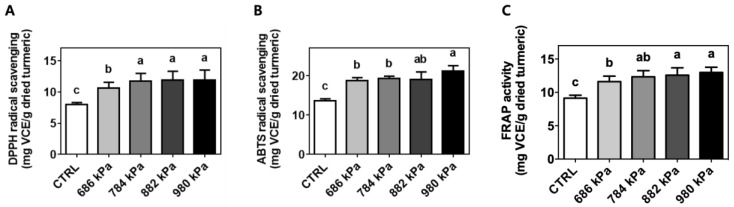
Enhanced antioxidant capacity of turmeric extract using puffing. Means with different letters (a–c) were statistically significant at *p* < 0.05 within the identified radical-scavenging assay (*n* = 4) of (**A**) DPPH radical scavenging, (**B**) ABTS radical scavenging, and (**C**) FRAP assay.

The antioxidant capacity of the control and puffed turmeric extracts was investigated using the well-accepted DPPH radical-scavenging, ABTS radical-scavenging, and FRAP assays [29]. Although the mechanisms of the antioxidant capacity assessment methods were different [30,31], the results showed the same patterns. Specifically, the DPPH radical-scavenging capacity of the turmeric extract was increased by puffing from 8.03 ± 0.27 for the control to 10.63 ± 0.92, 11.74 ± 1.24, 11.88 ± 1.42, and 11.89 ± 1.62 mg VCE/g dried turmeric for the samples created at 686, 784, 882, and 980 kPa, respectively (Figure 3A). Similar trends were observed in the ABTS and FRAP assays as demonstrated in Figure 3B,C, respectively. ABTS radical-scavenging activity increased from 13.69 ± 0.48 for the control to 18.71 ± 0.80, 19.26 ± 0.61, 19.03 ± 1.96, and 21.24 ± 1.32 mg VCE/g dried turmeric for the samples puffed at 686, 784, 882, and 980 kPa, respectively. The FRAP assay also detected the highest antioxidant capacity (12.98 ± 0.78 mg VCE/mg dried turmeric) in the extract of turmeric puffed at 980 kPa. The turmeric puffed at 0 (control), 686, 784, and 882 kPa exhibited antioxidant capacities of 9.14 ± 0.43, 11.61 ± 0.81, 12.33 ± 0.93, and 12.58 ± 1.09 mg VCE/g dried turmeric, respectively, as determined using FRAP.

### 3.5. Upregulation of Pro-Inflammatory Cytokine Production by Puffed Turmeric Extracts

**Figure 4:**
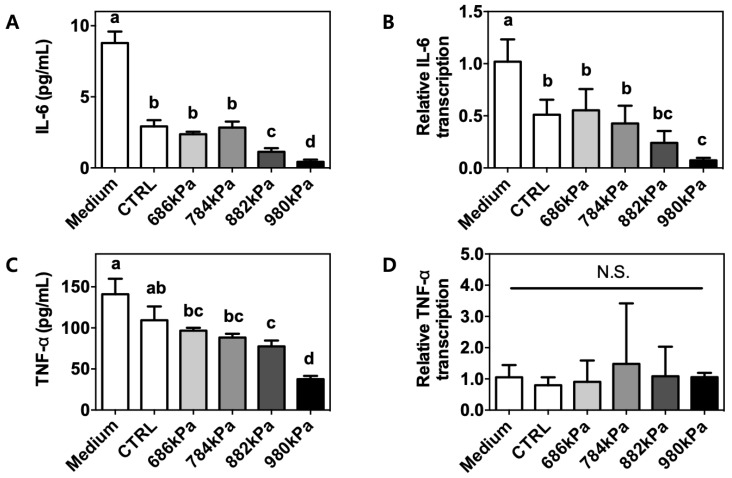
Reduced production of inflammatory cytokines in RAW264.7 macrophages following treatment with puffed turmeric extracts. Following treatment with 500 nm/mL LPS for 12 h to induce an inflammatory response, the culture media and cell pellets were separated for quantification of secreted cytokines (**A** and **C**) and intracellular mRNA levels (**B** and **D**), respectively. Means with different letters (a–d) were statistically significant at *p* < 0.05 within the panel (*n* = 4).

The culture supernatants of RAW264.7 cells stimulated with LPS in the presence of a turmeric extract were assessed for the presence of pro-inflammatory cytokines using commercially available ELISA kits. The anti-inflammatory effects of turmeric extract have previously been demonstrated [22] and were confirmed through increased media levels of IL-6 (8.78 ± 0.81 vs. 2.92 ± 0.44 pg/mL for the control, Figure 4A) and TNF-α (141.0 ± 18.78 vs. 109.40 ± 16.73 pg/mL for the control, Figure 4C). Interestingly, further reductions in IL-6 (2.37 ± 0.18, 2.84 ± 0.42, 1.14 ± 0.25, and 0.42 ± 0.16 pg/mL) and TNF-α (96.66 ± 3.50, 88.20 ± 4.68, 77.49 ± 7.08, and 37.63 ± 3.95 pg/mL) were observed in the presence of extracts of turmeric puffed at 686, 784, 882, and 980 kPa, respectively. The same trends were seen in the IL-6 mRNA level as determined using qRT-PCR (1.10 ± 0.21, 0.51 ± 0.14, 0.55 ± 0.20, 0.43 ± 0.17, 0.24 ± 0.11, and 0.07 ± 0.02 arbitrary units for the baseline, control, 686 kPa, 784 kPa, 882 kPa, and 980 kPa samples, respectively) (Figure 4B). In contrast, the TNF-α mRNA level was not affected (Figure 4D) by either the control or puffed turmeric extracts.

### 3.6. Suppression of Activation Markers on Macrophages Using Puffed Turmeric Extracts

**Figure 5:**
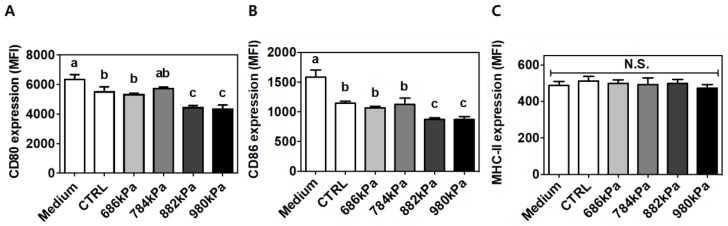
Regulation of activation-marker expression on RAW264.7 macrophages following treatment with puffed turmeric extracts. The expression of CD80 (**A**), CD86 (**B**), and MHC-II (**C**) was quantitatively assessed using staining with fluorescently labeled specific antibody followed by flow cytometry. Means with different letters (a–c) were statistically significant at *p* < 0.05 within the panel (*n* = 4). MFI—mean fluorescence intensity; N.S.—not significant.

Similar to the results of cytokine production, turmeric treatment reduced the expression of CD80 (MFI of 6339.88 ± 328.83 vs. 5493 ± 355.77 for the control) (Figure 5A) and CD86 (MFI of 1582.32 ± 121.17 vs. 1144.83 ± 32.77 for the control) (Figure 5B). The expression of CD80 (5310 ± 96.36, 5720.21 ± 110.47, 4424.44 ± 152.63, and 4323.11 ± 291.00 MFI) and CD86 (1068.93 ± 26.19, 1124.19 ± 110.29, 875.84 ± 26.98, and 871.84 ± 82.04 MFI) was further decreased as puffing pressure increased to 686, 784, 882, and 980 kPa, respectively. In contrast, MHC-II expression was not affected by either the control or puffed turmeric extracts (Figure 5C).

## 4. Discussion

In the current study, an easy and effective processing method for enhancing the antioxidant and anti-inflammatory activities of turmeric was sought. For this purpose, turmeric was puffed at various pressures, resulting in the volume expansion and darkened color depicted in Figure 1. In addition, turmeric puffed at a pressure of 784 kPa or higher exhibited tissue damage, indicating an increased internal vapor pressure. Colorimetric measurements quantitatively assessed the color change to confirm the darkening of turmeric powder using puffing (Figure 2). Ground turmeric exhibited a lower moisture content following puffing, as expected, since the puffing process involves heat- and pressure-induced vaporization [11]. Proximate analyses indicated that puffing did not affect the crude protein or crude ash content; however, a 19% increase in crude fat content was observed following puffing at 882 kPa (Table 1). These observations are slightly different from those of previous studies, in which the crude fat contents of coffee beans and cacao beans were decreased using puffing [9,11], indicating that the changes in proximate components depended on the target material. It seems that puffing at a higher pressure caused carbonization of turmeric, resulting in the reduced formation of porous structures and fat extraction. In previous studies on puffed ginseng or doraji, the extraction yield increased by up to 75% due to the increased permeability of the extracting solvent through the porous structure [11,12,14]. In contrast, the extraction yields of the control puffed turmeric samples were not affected, as described in Table 2.

Interestingly, puffing increased the TPC in the turmeric extracts by up to 53%, whereas the TFC was not affected. Similarly, it was previously reported that puffing effectively enhanced the TPC of jujube and coffee beans [11,32]. Han et al. also demonstrated augmented TPC and reduced TFC after explosive puffing in coffee beans compared to conventional roasting [33]. In contrast, however, the TPC of puffed cacao beans decreased with increasing puffing pressure [9], implying that TPC and TFC were differentially affected by puffing in various food materials. Overall, the data imply that puffing can be applied to turmeric for the enhancement of TPC. It has been shown that the brown-colored products of the Maillard reaction have an antioxidant capacity [34,35]. Puffed turmeric underwent the Maillard reaction, as evidenced by the obvious browning in color and increased TPC. Therefore, the antioxidant capacities of turmeric extracts were tested using three independent methods: DPPH radical-scavenging, ABTS radical-scavenging, and FRAP assays. The results showed increases of 48, 55, and 42%, respectively, after puffing in a pressure-dependent manner (Figure 3). These results are in accordance with previous reports in that the antioxidant effects of cacao or coffee beans were increased by puffing [9,11]. The increased amounts of Maillard reaction products and TPC in puffed turmeric might have contributed to these observations.

Macrophages play a key role in innate immunity as they cue inflammatory signals. In this regard, the murine cell line RAW264.7 is widely used for the screening of anti-inflammatory properties in natural products. Following LPS engagement with toll-like receptor (TLR) 4 on the surface of macrophages, intracellular signaling occurs via the myeloid differentiation primary response (MyD)88-nuclear fator (NF)κB axis. In brief, activated NFκB in the form of the v-rel avian reticuloendotheliosis viral oncogene homolog A (RelA)-p50 dimer penetrates the nucleus and initiates transcription of inflammatory genes including *IL-6* and *TNF-α*. To determine if puffing augmented the anti-inflammatory properties of turmeric, the secreted proteins and intracellular mRNA levels of IL-6 and TNF-α were quantified. Turmeric extract-treated cells exhibited reduced secretion of IL-6 and TNF-α compared to untreated cells (Figure 4A,C respectively). However, a reduction of cellular mRNA level was observed only for *IL-6* but not *TNF-α* (Figure 4B,D respectively). This discrepancy may be due to macrophage activation with LPS for 12 h in the current experimental setting. TNF-α is expressed within 2 h of LPS stimulation, but IL-6 production is initiated after 6 h [36], indicating that IL-6 was in the process of production and/or secretion at the time of measurement, but TNF-α had been fully secreted to the extracellular medium. It is also noteworthy that the concentration of TNF-α in the medium was approximately 10-fold different from that of IL-6 (Figure 4A,C, respectively). The puffing of turmeric enhanced the anti-inflammatory properties of the subsequent extract by up to 96 and 73% for IL-6 and TNF-α, respectively, in a pressure-dependent manner. Macrophages can also be assessed for their activation status through the quantification of the expression of CD80, CD86, and MHC-II on the cellular surfaces [20,28]. LPS-stimulated RAW264.7 cells in the presence of either a control or puffed turmeric extract were incubated with specific antibodies conjugated with a fluorochrome. Following a series of washing steps, the cells were resuspended and analyzed using flow cytometry. The expression of each surface marker was quantified using the fluorescence intensity of the specific marker and calculated as the mean fluorescence intensity (MFI, arbitrary units). Similar to cytokine production, expression of CD80 and CD86 (Figure 5A,B, respectively) was reduced in cells treated with non-puffed turmeric extract compared to no treatment. In addition, treatment with puffed turmeric extracts further downregulated the expression of CD80 and CD86, confirming the augmented anti-inflammatory properties of puffed turmeric. In contrast, MHC-II expression was not affected by treatment with either the control or puffed turmeric extracts (Figure 5C). Because MHC-II is an antigen-presenting molecule to T-lymphocytes for the activation of adaptive immune responses [20], the current results imply that puffing of turmeric contributes to the downregulation of inflammatory responses but does not weaken activation of adoptive immunity.

## 5. Conclusions

Overall, the current study shows for the first time that puffing is a promising method for turmeric processing. Due to an increase in the Maillard reaction and extraction of TPC following puffing, the antioxidant and anti-inflammatory properties of the extracts were significantly enhanced. Additional studies for the assessment of bioavailability and sensory evaluation are required for the wider application of turmeric as a food ingredient.

## Figures and Tables

**Table 1:** Changes in proximate analysis induced by puffing (*n* = 3).

Puffing Pressure	Moisture Content (%, w.b.^1^)	Crude Fat (% Dried Turmeric)	Crude Protein (% Dried Turmeric)	Crude Ash (% Dried Turmeric)
Control	15.69 ± 0.09 ^a,2^	6.39 ± 0.14 ^b^	7.73 ± 0.06 ^a^	6.53 ± 0.36 ^a^
686 kPa	11.57 ± 0.41 ^b^	6.79 ± 0.70 ^a,b^	8.47 ± 0.73 ^a^	6.64 ± 0.7 ^a^
784 kPa	12.06 ± 0.69 ^b^	7.46 ± 1.27 ^a^	8.49 ± 0.32 ^a^	6.52 ± 0.93 ^a^
882 kPa	11.54 ± 0.56 ^b^	7.57 ± 0.69 ^a^	8.17 ± 0.91 ^a^	6.93 ± 0.47 ^a^
980 kPa	10.74 ± 1.22 ^c^	6.91 ± 0.81 ^a,b^	7.81 ± 0.61 ^a^	6.36 ± 0.79 ^a^

^1^ w.b.—wet based including water content in total weight. ^2^ Values designated by different letters (^a–b^) are statistically different at *p* < 0.05 within the column.

**Table 2:** Extraction yield, TPC, and TFC (*n* = 3).

Puffing Pressure	Extraction Yield (%, w/w)	Total Phenolic Content (mg GAE/g Dried Turmeric)	Total Flavonoid Content (mg CE/g Dried Turmeric)
Control	10.82 ± 0.80 ^a,1^	8.81 ± 0.81 ^d^	9.66 ± 0.63 ^a^
686 kPa	11.33 ± 1.71 ^a^	11.55 ± 1.14 ^c^	10.34 ± 0.53 ^a^
784 kPa	11.10 ± 0.70 ^a^	12.28 ± 0.84 ^b^	10.46 ± 1.10 ^a^
882 kPa	10.82 ± 1.00 ^a^	12.93 ± 0.83 ^a,b^	10.45 ± 0.83 ^a^
980 kPa	10.85 ± 0.94 ^a^	13.49 ± 0.66 ^a^	9.86 ± 0.54 ^a^

^1^ Values designated by different letters (^a–d^) are statistically different at *p* < 0.05 within the column.
